# News and Notes

**Published:** 1904-10

**Authors:** 


### *NEWS AND NOTES.*

WORK has begun on the new $50,000 quarantine hospital at Buffalo.

DR. WM. OSLER has been elected Regius Professor of Medicine at Oxford, England.

SIXTY new cases of typhoid fever were reported to the Health Department of Cincinnati last week.

ROBERT KOCH will retire October 1 from the position of director of the Institute for Infectious Diseases at Berlin.

SIR JOHN SIMON, K.C.B., former president of the Royal College of Surgeons and of the Royal Society, died in London on July 23.

M. DOYEN, the well known surgeon, is a candidate for a seat in the Chamber of Deputies as representative of one of the arrondissements of Paris.

ANTOIN CHECHOFF died at Badenweiler, Germany, on July 15. He was a physician, but his reputation throughout Russia was as a literary man.

THE fifth annual meeting of the American Roentgen Ray Society was held September 9, 10, 12 and 13, 1904, at the Louisiana Building, St. Louis, Mo.

THE degree of doctor of laws was recently conferred upon Dr. William Osler by Harvard University, and upon Dr. William Halsted by Yale University.

DR. GEORGE F. SHRADY, after nearly forty years of continuous service, has resigned the editorship of the *Medical Record*, Dr. Thos. L. Stedman being appointed in his place.

---

THE Fourth Pan-American Medical Congress, which was to have convened the latter part of December of this year at Panama, has been postponed until the first week in January.

---

A DISPATCH from Austin says that the Texas State Health Department has been informed of the breaking out of yellow fever at the Government military post at Brownsville, Tex.

---

AT the conference arranged by the Chicago Woman's Club on "Women in Industrialism," Dr. Lucy Waite, of Chicago, reported 4,376 women listed in the medical societies of this country.

---

ACCORDING to a statement issued by Dr. Edward Martin, Director of the Department of Public Health and Charities, the city of Philadelphia is, for the first time in three years, free from smallpox.

---

THE list of notifiable diseases has recently been revised and enlarged by the Board of Health of Philadelphia. The list now includes cholera, yellow fever, malarial fever, typhoid fever, typhus fever, scarlet fever, smallpox, chickenpox, diphtheria, cerebrospinal meningitis, measles, rubella, whooping-cough, tuberculosis, pneumonia, erysipelas, puerperal fever, plague, trachoma, leprosy, glanders, tetanus, rabies and anthrax.

---

THE fortieth anniversary of the introduction of subcutaneous injections of calomel in the treatment of syphilis was celebrated recently in Italy. A committee consisting of Drs. Domenico Maiocchi, Ambrogio Bertarelli, and Mario Truffin, with Professor

Camillo Golgi, Rector of the University of Pavia, as honorary president, presented a gold medal to Dr. Angelo Scarenzio, to whom he is attributed the invention of the method.

---

THE Royal Colleges of Physicians and Surgeons of Ireland have added Irish to the list of optional languages, one of which must be taken by each candidate at the preliminary examination held by the colleges. The following resolution, adopted at a recent meeting of the Kilkenny branch of the Gaelic League, makes the action of the college more obvious: "*Resolved*, That we will call on our elected representatives at the board of guardians to direct their employees to at once learn to speak the language of the Gael, and that Drs. Hackett and Morris write their prescriptions also in the Irish language under the pain of dismissal." Naturally the colleges did not want their future licentiates to be subject to instant dismissal for such gross ignorance.

---

THE first International Congress on the Sanitation of the Dwelling-house, organized under the auspices of the French Society of Hygiene, will be held at Paris, October 15 to 20, 1904, under the presidency of M. Janssen, member of the Institute. Among the honorary presidents, in addition to the leading hygienists of France, are the Prime Minister and the Ministers of Public Instruction, Agriculture, Commerce, and Marine. The object of the congress is to study the sanitary conditions of the building and installation of dwelling-houses, to urge the reforms in these conditions that appear to be required, and to discuss the practical means of inducing municipalities, owners, architects, engineers, builders and tenants to apply the principles of hygiene to the construction of dwelling-houses.

